# Elevated Serum Interleukin-18 Level Is Associated with All-Cause Mortality in Stable Hemodialysis Patients Independently of Cardiac Dysfunction

**DOI:** 10.1371/journal.pone.0089457

**Published:** 2014-03-05

**Authors:** Yen-Wen Liu, Chi-Ting Su, Yu-Tzu Chang, Wei-Chuan Tsai, Yu-Ru Su, Saprina P. H. Wang, Chun-Shin Yang, Liang-Miin Tsai, Jyh-Hong Chen, Junne-Ming Sung

**Affiliations:** 1 Division of Cardiology, Department of Internal Medicine, National Cheng Kung University Hospital, College of Medicine, National Cheng Kung University, Tainan, Taiwan; 2 Division of Nephrology, Department of Internal Medicine, National Taiwan University Hospital Yun-Lin Branch, Yun-Lin, Taiwan; 3 Division of Nephrology, Department of Internal Medicine, National Cheng Kung University Hospital, College of Medicine, National Cheng Kung University, Tainan, Taiwan; 4 Department of Statistics, National Cheng Kung University, Tainan, Taiwan; 5 Graduate Institute of Biomedical Engineering, National Cheng Kung University, Tainan, Taiwan; 6 Division of Nephrology, Department of Internal Medicine, Catholic Fu-An Hospital, Yun-Lin, Taiwan; Centre Hospitalier Universitaire Vaudois, Switzerland

## Abstract

**Background:**

High circulating interleukin (IL)-18 level predicts a higher hospitalization rate among dialysis patients, possibly through cardiovascular mechanisms; however, whether higher IL-18 level is associated with mortality in dialysis patients is less clear. In addition, its impacts on left ventricular (LV) function are also unknown. We conducted a cohort study to examine the impacts of IL-18 level on LV function and prognosis among clinically stable hemodialysis patients.

**Methods:**

Clinically stable patients undergoing maintenance hemodialysis (≥3 months) were prospectively enrolled from December 2008 to January 2009, and were followed up for 31 months. The enrolled patients (41% male, 66.4±10.9 years of age) received 2-dimensional echocardiography and myocardial deformation (strain) analysis, including LV peak systolic longitudinal strain (GLS) and circumferential strain (CS). Laboratory measurements were also performed. Cox regression analysis was used to investigate prognostic factors.

**Results:**

Seventy-five patients were stratified into 2 groups by the median value of IL-18 (654.2 pg/ml). Between these 2 groups, there was no significant difference in baseline characteristics including LV ejection fraction. The high IL-18 group had a worse LV systolic function as demonstrated by reduced GLS and CS. Seventeen patients (22.7%) died during the follow-up period. Multivariate Cox regression analysis showed that low serum albumin, the presence of hypertension, high serum IL-18, and less negative GLS (>−15%) were independently associated with all-cause mortality. No significant interaction between IL-18 and less negative GLS was noted in the final Cox model.

**Conclusion:**

Hemodialysis patients with high IL-18 levels tend to have worse LV systolic function and higher mortality rate. However, elevated serum IL-18 level is predictive of poor prognosis among stable hemodialysis patients, independently of LV dysfunction. This suggests an additional value of IL-18 to echocardiographic study in predicting all-cause mortality, and IL-18 may be helpful in early risk stratification of hemodialysis patients.

## Introduction

End-stage renal disease (ESRD) is notorious for high mortality, and cardiovascular disease is the leading cause of morbidity and mortality [1]–[3]. Thus, early cardiovascular risk stratification and understanding the mechanism(s) are important issues in managing dialysis patients, and they may enable early identification of high-risk patients and optimizing therapeutic interventions. Increased circulating levels of pro-inflammatory cytokines, such as C-reactive protein (CRP), interleukin (IL)-6 and 18, can be detected among ESRD patients. The increased pro-inflammatory cytokines play a crucial role in chronic inflammation, and are associated with cardiovascular events and poor outcomes in dialysis patients [4]–[7]. Among the inflammatory cytokines, elevated IL-18 level was shown to be associated with higher future hospitalization rate in dialysis patients, possibly, through cardiovascular mechanisms [7], [8]. Evidence from the experimental and clinical studies emerge that the expression of IL-18 is intimately related to atherosclerotic plaque progression and vulnerability [9]–[12]. Furthermore, overexpression of IL-18 was reported to lead to aggravated cardiac remodeling in animals [13], and daily administration of IL-18 may cause myocardial dysfunction in healthy mice [14]. Thus, it is suggested that IL-18 possibly causes LV dysfunction indirectly by aggravating coronary atherosclerosis or directly by acting on cardiomyocytes to induce myocardial dysfunction [11]–[14]. Increased levels of circulating IL-18 have proved to be a strong and independent predictor of cardiovascular death in patients with coronary artery disease (CAD) [15]. However, whether a higher IL-18 level is associated with mortality and whether IL-18 is useful for early risk stratification in dialysis patients are still unclear.

Cardiac structural and functional abnormalities are associated with high cardiovascular and all-cause mortality among ESRD patients, and cardiovascular abnormalities can be used for risk stratification of hemodialysis patients. [16]–[18]. To explore the role of IL-18 in all-cause mortality, the relationships between the serum IL-18 level and outcome-related cardiac structural and functional abnormalities should be clarified. Left ventricular (LV) function in ESRD patients has been studied extensively by conventional echocardiographic parameters, e.g. LV ejection fraction (LVEF). However, these measurements are semi-quantitative and are insensitive in the early detection of subtly deteriorating cardiac function [19]. Two-dimensional speckle-tracking echocardiography (STE) with myocardial deformation analysis (2D strain analysis) is an objective and reproducible modality that is more sensitive than conventional echocardiographic study for assessing subtle LV dysfunction, and it is especially true for evaluating the systolic function [20]–[22]. LV peak systolic longitudinal strain (GLS) (or circumferential strain [CS]) is the ratio of the maximal change in myocardial longitudinal (or circumferential length) in systole to the original length. During systole, LV myocardium in the longitudinal and circumferential directions shortens, so GLS and CS are represented by a negative value. More negative of GLS or CS values refer to better LV systolic function. Importantly, less negative GLS has been proven to be a more sensitive and powerful predictor of all-cause mortality than LVEF in general population [23]. Furthermore, our recent study indicated an additional value of GLS to conventional echocardiography in predicting all-cause and cardiac mortality in stable hemodialysis patients with preserved LVEF (LVEF≥50%) [24]. To date, the impact of elevated IL-18 on LV function is not well studied in ESRD patients receiving maintenance hemodialysis. In the current studies, we aimed to investigate the association between IL-18 levels and LV function using 2D strain analysis and to assess the outcome predictive effects of IL-18 in clinically stable hemodialysis patients.

## Materials and Methods

### Ethics Statement

The study adhered to the Declaration of Helsinki. The study protocol was approved by the Human Research and Ethics Committee of the National Cheng Kung University Hospital (IRB number: ER-98-073). All the enrolled patients provided written informed consent.

### Study Design

As previously described [24], [25], from December 2008 to January 2009, we prospectively enrolled adult ESRD patients (≥18 years old) receiving maintenance hemodialysis program, 4 hours/day, three times a week for more than 3 months, from two community hospitals in Yun-Lin, Taiwan: the National Cheng Kung University Hospital Dou-Liou Branch, and the Catholic Fu-An Hospital. The exclusion criteria are as follows: (1) severe valvular heart disease (including mitral/aortic regurgitation or stenosis); (2) recent infection; (3) hospitalization due to an active episode of decompensated heart failure (HF) presenting with pulmonary edema (≥NYHA FC III) or acute coronary syndrome in recent 3 months; (4) atrial fibrillation; (5) refusing blood tests; and (6) poor echocardiographic image quality.

Upon enrollment, clinical information on co-morbidities, medical history, and current cardiovascular medication were obtained by careful review of each patient's medical record and a self-reported questionnaire. Patients' compliance with regards to prescribed medication was reliably ascertained. Additionally, the adequacy of dialysis was evaluated based on the recommendations of the Kidney/Disease Outcome Quality Initiative (KDOQI). The enrolled patient medical records during the follow-up period (31 months, from December 2008 to June 2011) were carefully reviewed, and all the participants were assured to receive adequate clearance of dialysis.

### Laboratory Measurements

Blood was collected just before the midweek dialysis session in the same week when echocardiographic study was performed. Serum was stored at −80°C until analysis and thawed to measure the levels of high-sensitivity C-reactive protein (hsCRP, BN II analyzer; Dade Behring, Glasgow, DE), IL-6 (chemiluminescent sandwich ELISA, Quantikine Human IL-6; R&D Systems Inc., Minneapolis, MN, USA), IL-18 (Sandwich ELISA, R&D Inc., Minneapolis, MN, USA), and procollagen type I C-terminal peptide (PICP, Takara Bio Inc., Otsu, Shiga, Japan) [24], [25]. Serum cholesterol, triglyceride, calcium, phosphate, and albumin were measured using an automatic analyzer.

### Echocardiographic Measurements

All patients were examined in the left lateral decubitus position by one well-trained cardiologist (YWL, with 10 years' experience with echocardiographic examination) using a commercially available ultrasound system with a 3.5 MHz probe (Vivid-i, GE Healthcare, Horten, Norway). As previously described, two-dimensional STE and tissue Doppler imaging (TDI) were obtained [21], [22], [24], [25]. All hemodialysis patients received an echocardiographic examination during the halfway point of the hemodialysis session (the second or third hour of each session) [22]. According to the recommendation of the American Society of Echocardiography (ASE) [26], [27], we measured LV mass index, volume, and LVEF, left atrial volume index. Using pulsed-wave Doppler, we measured the peak early (E)-wave and late (A)-wave velocities of the mitral inflow.

The pulse TDI of the mitral annulus movement was acquired from the apical 4-chamber view when a sample volume was placed first at the septal side and then at the lateral side of the mitral annulus. To obtain the peak systolic (s′) and early diastolic (e′) velocities, we measured 3 end-expiratory beats and averaged these values for further analysis. We used the average e′ velocity acquired from the septal and lateral sides of mitral annulus to calculate ratio of the mitral inflow E velocity to e′ velocity (average E/e′ = E/[(e′_septal_+e′_lateral_)/2]). We acquired 2D gray-scale images in the 3 standard apical views (i.e. apical 4-chamber, apical 2-chamber, and apical 3-chamber) for 3 cardiac cycles and stored digitally with a frame rate of 50–90 frames/second for subsequent off-line analysis.

### Inferior vena cava (IVC) diameter measurement in ESRD patients

To evaluate the fluid status of ESRD patients, we measured IVC diameter twice at the end of expiration in a subxiphoid location and just proximal to the junction of the hepatic veins that lie approximately 0.5 to 3.0 cm proximal to the ostium of the right atium [22], [28]–[30]. The average value of the measured end-expiratory IVC diameter was defined as IVCe. It has been reported that IVCe>1.53 cm is a marker of hypervolemia in ESRD patients [22], [30].

### Echocardiographic Analysis

Off-line 2D strain analysis was performed using automated function imaging software (EchoPAC work station, BT09, GE Healthcare, Israel). Peak systolic longitudinal strain was automatically obtained from the 3 standard apical views. The average peak systolic longitudinal strain value from the 3 apical views was regarded as GLS [21], [22], [24]. Six LV segments on the para-sternal short-axis view at the mid-papillary level were examined to obtain the circumferential strain in systole and the average of the circumferential strain of these six segments was defined as CS [21], [24].

Because the hemodialysis patients received echocardiographic examinations during dialysis in the study, we performed echocardiography twice (during hemodialysis and on a non-dialysis day) for 10 of these patients to explore whether hemodialysis per se affects GLS. There was no significant difference in GLS during hemodialysis versus a non-dialysis day (−17.0±5.8% *vs.* −16.6±5.8%, respectively; *p* = 0.87). In addition, the Bland-Altman analysis revealed no systemic bias of GLS between intra- and inter-observer agreements [24].

### Statistical Analysis

Continuous data were presented as mean ± standard deviation. Dichotomous data were presented as numbers and percentages. The differences in continuous variables were evaluated using Student's *t*-test, or Mann-Whitney U test when the data did not follow normal distributions. Chi-square test or Fisher's exact test was used for comparing categorical variables. Kaplan-Meier method with log-rank test was used to compare survival rates between strata. The relationships among continuous variables were evaluated using Pearson correlation analysis or Spearman's correlation analysis when the data did not follow normal distributions. The uni- and multi-variate Cox regression analyses were used to examine the risk factors of all-cause mortality. A *p*<0.05 was considered statistically significant, and all statistical analyses were made using the SPSS (Statistical Package for the Social Sciences) software (version 17.0, SPSS Inc, Chicago, IL, USA).

## Results

### Clinical Characteristics

In total, 109 ESRD patients undergoing maintenance hemodialysis were eligible for this study. Patients were subsequently excluded because of old age (≥80 years, n = 3), atrial fibrillation (n = 4), recent infarction (n = 1), decompensated HF within 3 months (n = 5), severe valvular heart disease (n = 2), or inadequate image quality for analysis (n = 6). Thirteen patients were subsequently excluded due to missing data of IL-18 (because of insufficient volumes of stored serum in theses 13 patients for assaying IL-18 levels); thus, there were 75 patients entering the final statistic analyses. There was no significant difference, except for modest difference of cholesterol and triglyceride levels, between the baseline clinical characteristics of patients included in final analyses and those of 19 excluded patients (6 with inadequate echocardiographic images and 13 with missing IL-18 data). Furthermore, there was no significant difference in echocardiographic parameters between patients included in final analyses and 13 excluded because of missing IL-18 data. All enrolled patients presented with anuria. Based on the recommendations of the KDOQI, they all received adequate hemodialysis (average Kt/V [an indicator of dialysis adequacy; K, urea clearance; T, dialysis time; V, urea distribution volume] was 1.71±0.24; and cardiothoracic ratio on chest X-ray was 48±5%).

Since the distribution of IL-18 levels was skewed to the right (skewness = 2.14), we defined the median concentrations of IL-18 (654.2 pg/ml) as the cut-off values. According to this cutoff, the enrolled patients were stratified into two groups: (1) high IL-18 group (IL-18 ≥654.2 pg/ml, n = 37) with IL-18 values of 1479.5±753.5 pg/ml; and (2) low IL-18 group (IL-18 <654.2 pg/ml, n = 38) with IL-18 values of 432.6±131.2 pg/ml. There was no significant difference of demographic data, concomitant diseases (except chronic hepatitis), medication(s), and blood biochemical test results between these two groups (Table 1).

**Table 1:** Baseline demographic characteristic of hemodialysis (HD) patients.

	Total (n = 75)	IL-18 <654.2 pg/ml (n = 38)	IL-18 ≥654.2 pg/ml (n = 37)	*p* ★
Age (years)	66.4±10.9	66.4±11.1	66.4±10.9	0.97
Male, n (%)	31 (41%)	16 (42%)	15 (41%)	0.89
BMI (kg/m^2^)	21.9±2.8	21.8±3.1	22.0±2.5	0.76
Heart Rate (beats/minute)	77.1±12.1	76.8±13.3	77.5±10.9	0.81
SBP (mmHg)	146.1±16.0	145.3±16.4	147.0±15.9	0.65
DBP (mmHg)	76.9±8.5	75.9±7.1	77.9±9.6	0.30
HD duration (years)‡	6.1±5.1	5.0±3.7	7.4±6.2	0.24
Kt/V	1.70±0.24	1.74±0.26	1.67±0.21	0.22
IDWG (%)	5.2±1.8	4.9±1.5	5.5±2.1	0.18
**Concomitant diseases, number (%)**				
Prevalent CAD	30 (40%)	12 (32%)	17 (46%)	0.20
Heart failure under control	12 (16%)	5 (13%)	7 (19%)	0.50
Diabetes mellitus	38 (51%)	18 (47%)	20 (54%)	0.56
Hypertension	64 (85%)	32 (84%)	32 (86%)	0.78
Hyperlipidemia	20 (27%)	11 (29%)	9 (24%)	0.65
LV hypertrophy	60 (80%)	29 (76%)	31 (84%)	0.67
Chronic hepatitis	38 (51%)	14 (37%)	24 (65%)	0.02
**Cardiovascular drugs, number (%)**				
CCB	41 (55%)	20 (53%)	21 (57%)	0.72
β-Blockade	34 (45%)	17 (45%)	17 (46%)	0.92
ACEIs/ARBs	42 (56%)	21 (55%)	21 (57%)	0.90
Statin	12 (16%)	5 (13%)	7 (19%)	0.50
**Serum biochemistry study**				
Calcium (mg/dL)	9.2±0.8	9.3±0.8	9.1±0.8	0.45
Phosphate (mg/dL)	4.5±1.3	4.5±1.3	4.5±1.4	0.87
Albumin (g/dL)	3.3±0.3	3.4±0.3	3.3±0.4	0.17
Cholesterol (mg/dL)	167.2±38.6	169.6±36.6	164.8±40.8	0.60
Triglyceride (mg/dL)‡	143.9±114.0	156.4±144.7	131.3±71.4	0.91
IL-6 (pg/ml)‡	20.1±59.5	27.3±81.9	12.2±9.0	0.76
IL-18 (pg/ml)‡	949.1±750.0	731.6±550.5	1172.4±862.3	<0.001
hsCRP (mg/dL)‡	1.0±1.5	0.5±0.5	1.5±2.0	0.03
PICP (ng/ml)	878.5±382.5	842.8±356.8	917.4±410.3	0.41

**Abbreviations:** ACEI, angiotensin-converting enzyme inhibitor; ARB, angiotensin II-receptor blocker; BMI, body mass index; CAD, coronary artery disease; CCB, calcium channel blocker; DBP, diastolic blood pressure; hsCRP, high-sensitivity C-reactive protein; IDWG, inter-dialytic weight gain; IL, interleukin; LV, left ventricular; PICP procollagen type I C-terminal peptide.; SBP, systolic blood pressure.

Continuous data are expressed as mean ± SD.

‡ Non-normal distributed continuous data.

^^★^^ *p* value by Student's *t*-test for normal distributed continuous data, nonparametric Mann-Whitney U test for non-normal distributed continuous data, and chi-square test or Fisher's exact test for categorical variables.

### Follow-up outcome

**Figure 1:**
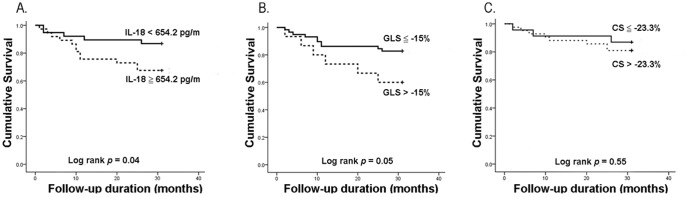
Kaplan-Meier survival curves of all-cause mortality in clinically stable hemodialysis patients using the cutoff values of interleukin (IL)-18 (A), left ventricular global peak systolic longitudinal strain (GLS) (B), and circumferential strain (C).

The enrolled patients were followed up for 26.4±9.4 months (range 1 to 31 months). No patient was lost during the follow-up period. Seventeen patients (22.7%) died, including eight from cardiovascular death, six from infections, and three from liver diseases. Importantly, the high IL-18 group had a worse prognosis (mortality rate: high IL-18 group *vs.* low IL-18 group: 32.4% *vs.* 13.2%, *p* = 0.046, Figure 1A).

### Evaluation of Volume Status

In this study, LVEDVi (LV end-diastolic volume index) and IVCe were measured to assess the patients' volume status. There was no significant difference in either LVEDVi or IVCe between the two groups (Table 2), and the IVCe was not engorged in either group. These data indicated that the patients included in these two groups were not hypervolemic.

**Table 2:** Echocardiographic study results of stable hemodialysis patients.

	Total (n = 75)	IL-18 <654.2 pg/ml (n = 38)	IL-18 ≥654.2 pg/ml (n = 37)	*p* ★
**Traditional parameters**				
LV EDVi (ml/m^2^)‡	73.6±25.8	72.4±23.6	74.5±27.7	0.78
LV mass index (gm/m^2^)‡	153.2±58.1	153.5±61.3	152.8±49.5	0.46
LV EF (%)‡	62.9±8.8	63.6±8.6	62.1±9.1	0.68
E velocity (cm/sec)	80.0±29.6	74.8±27.7	85.8±31.0	0.13
E/A	0.82±0.40	0.87±0.50	0.75±0.22	0.19
LAVi (ml/m^2^)	34.8±8.1	32.9±8.3	36.3±7.8	0.16
IVCe diameter (cm)	1.27±0.31	1.26±0.26	1.29±0.36	0.70
**Tissue Doppler imaging parameters**				
s′ (cm/sec)	8.3±2.2	8.5±2.2	8.0±2.2	0.32
Average e′ (cm/sec)	4.8±1.4	4.8±1.3	4.8±1.4	0.88
Average E/e′	17.5±9.1	17.0±10.5	18.1±7.4	0.63
**Two-dimensional strain analysis**				
GLS (%)	−17.8±3.9	−18.7±4.1	−16.8±3.4	0.03
LSRs (sec^−1^)	−0.97±0.22	−1.00±0.22	−0.92±0.22	0.13
Circumferential strain (%)	−20.6±6.3	−22.1±6.2	−18.8±6.0	0.03
CSRs (sec^−1^)	−1.90±0.65	−1.91±0.49	−1.88±0.80	0.85

Abbreviations: CSRs, systolic circumferential strain rate; EDVi, end-diastolic volume index; EF, ejection fraction; E/e′, early transmitral velocity to tissue Doppler mitral annular early diastolic velocity ratio; GLS, global left ventricular peak systolic longitudinal strain; IVCe, end-expiratory inferior vena cava diameter; LAVi, left atrial volume index; LSRs, systolic longitudinal strain rate; LV, left ventricular; s′, peak systolic mitral annular velocity.

Data are expressed as mean ± SD.

‡ Non-normal distributed continuous data.

^^★ *p* value by Student's *t*-test for normal distributed continuous data and nonparametric Mann-Whitney U test for non-normal distributed continuous data.^^

### Evaluation of Cardiac Function

Based on the diagnostic criteria of LV hypertrophy [26], most of the enrolled patients had LV hypertrophy (Tables 1 and 2). There were 4 patients with reduced LVEF (<50%) in the high IL-18 group and 2 in the low IL-18 group. No significant difference of LVEF or s′ existed between these two groups. Nevertheless, the high IL-18 group had less negative GLS and CS, indicating worse LV systolic function (Table 2), and this difference might suggest that subclinical LV systolic dysfunction may partially contribute to a worse prognosis in clinically stable hemodialysis patients with high IL-18 levels. In addition, most of the enrolled patients in both groups had LV diastolic dysfunction, presenting with reverse E/A ratio, high average E/e′ values, and increased PICP levels, indicating the presence of increased LV filling pressure [25].

Because GLS might be affected by the intravascular volume status or blood pressure, we evaluated patient characteristics by stratifying the patients into two groups (GLS≤−15% vs. GLS>−15%, Table 3). There was no significant difference in either blood pressure or hypertension prevalence, nor were these two patient groups hypervolemic.

**Table 3:** Baseline demographic characteristic of hemodialysis patients with left ventricular global peak systolic longitudinal strain (GLS)≤−15% or GLS>−15%.

	GLS≤−15% (n = 59)	GLS>−15% (n = 16)	*p*
Age (years)	67.0±10.5	64.1±12.6	0.40
Male, n (%)	22 (54%)	9 (56%)	0.17
BMI (kg/m^2^)★	22.0±2.9	21.4±2.5	0.61★
Heart Rate (beats/minute)	77.3±11.8	76.1±14.1	0.78
SBP (mmHg)	147.1±15.1	142.6±19.1	0.41
DBP (mmHg)	77.1±8.2	76.2±9.6	0.75
Hemodialysis duration (years)★	6.3±5.2	5.0±4.6	0.36★
Kt/V	1.72±0.22	1.64±0.31	0.43
IDWG (%)	5.1±1.9	5.4±1.4	0.54
Prevalent CAD	16 (27%)	13 (82%)	<0.01
Heart failure under control	4 (7%)	8 (50%)	<0.01
Diabetes mellitus	29 (49%)	9 (56%)	0.62
Hypertension	51 (86%)	32 (82%)	0.69
Hyperlipidemia	18 (31%)	2 (13%)	0.21
LV hypertrophy	50 (85%)	15 (94%)	>0.99
LV EDVi (ml/m^2^)	70.1±18.4	84.6±40.7	0.23
LV mass index (gm/m^2^)★	139.1±31.0	201.1±95.0	<0.01★
LV EF (%)	65.0±6.5	55.4±11.6	<0.01
E/A	0.78±0.37	0.96±0.46	0.17
LAVi (ml/m^2^)	33.3±7.7	41.6±6.8	0.01
IVCe diameter (cm)	1.21±0.23	1.50±0.44	0.03
s′ (cm/sec)	8.7±1.9	6.7±2.6	0.01
Average e′ (cm/sec)	5.0±1.3	4.0±1.3	0.01
Average E/e′★	16.3±8.0	21.9±11.6	0.03★
PICP (ng/ml)	877.0±394.2	883.9±349.3	0.95

Abbreviations: BMI, body mass index; CAD, coronary artery disease; DBP, diastolic blood pressure; EDVi, end-diastolic volume index; EF, ejection fraction; E/e′, early transmitral velocity to tissue Doppler mitral annular early diastolic velocity ratio; GLS, global left ventricular peak systolic longitudinal strain; IDWG, inter-dialytic weight gain; IVCe, end-expiratory inferior vena cava diameter; Kt/V, dialysis clearance; LAVi, left atrial volume index; LSRs, systolic longitudinal strain rate; LV, left ventricular; PICP procollagen type I C-terminal peptide; SBP, systolic blood pressure; s′, peak systolic mitral annular velocity.

Data are expressed as mean ± standard deviation or number (%).

^★^ *P* values were calculated by nonparametric Mann-Whitney U test.

### Prognostic predictors

**Figure 2:**
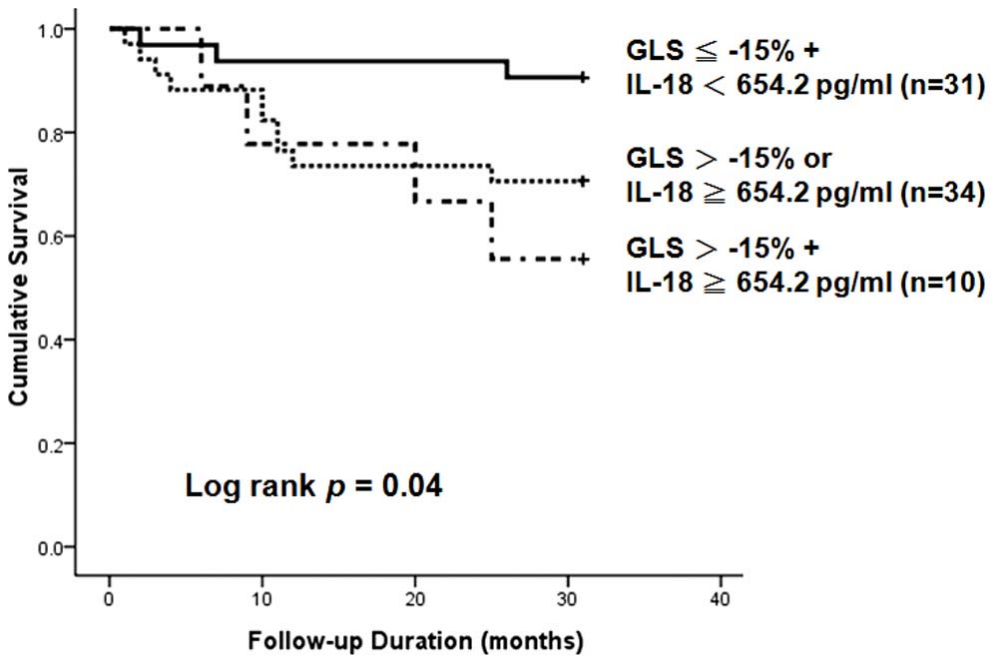
Kaplan-Meier survival curves of all-cause mortality in clinically stable hemodialysis patients using the cutoff values of interleukin (IL)-18 and left ventricular global peak systolic longitudinal strain (GLS). Clinically stable hemodialysis patients with high IL-18 levels (≥667 pg/ml) and less negative GLS (>−15%) had the worst prognosis.

Kaplan-Meier survival curve revealed that the high IL-18 group had higher all-cause mortality rate (Figure 1A). Recently, we proved that less negative GLS (GLS>−15%) is a prognostic predictor in clinically stable hemodialysis patients with preserved LVEF [24]. However, the cutoff of CS to evaluate its prognostic role in hemodialysis patients is still unknown. Based on a recent meta-analysis study regarding the normal value of CS [31], we defined the CS cutoff point as −23.3%. Using the cutoffs of GLS (−15%) and CS (−23.3%), we demonstrated that GLS (*p* = 0.05, nearly significant), but not CS (*p* = 0.55), might provide prognostic information (Figure 1B and 1C). Furthermore, patients with both a high IL-18 level and less negative GLS had the highest all-cause mortality rate (Figure 2), suggesting that the combination of IL-18 and GLS may be a more powerful prognostic predictor. However, these data cannot exclude the possible interaction between IL-18 level and GLS measurement to all-cause mortality in stable hemodialysis patients.

**Figure 3:**
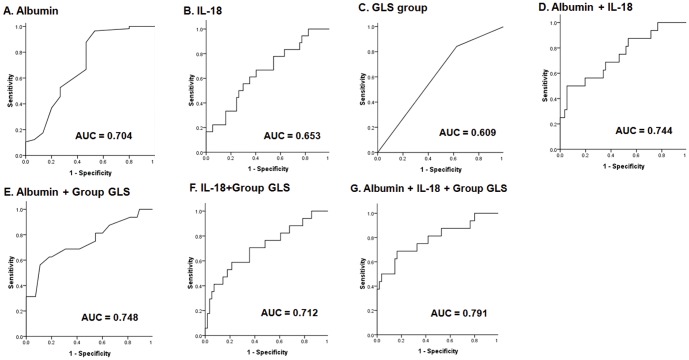
Receiver operating characteristic (ROC) curve of the ability of serum albumin (A), serum interleukin (IL)-18 (B), left ventricular global peak systolic longitudinal strain (GLS) group (C), the 2-combination parameters (D–F), and the 3-combination parameter (G) to predict all-cause mortality in clinical stable hemodialysis patients.

Subsequently, using univariate Cox regression analysis, we found several potential factors associated with the risk of all-cause mortality, including old age, prevalent CAD, hypertension, low serum albumin level, and increased serum IL-18 concentrations, and less negative GLS (all *p*<0.05 except for the reduced GLS [*p* = 0.05, marginal significance], Table 4). We then performed correlation analyses between different parameters of potential prognostic factors and found a modest correlation between IL-18 and hsCRP levels, and a moderate correlation between hsCRP and albumin levels (Table 5). We then conducted 5 multivariate Cox regression models to determine independent prognostic predictors of all-cause mortality. For the stable hemodialysis patients, decreased albumin level, hypertension, increased serum IL-18 level, and less negative GLS were the independent prognostic predictors (Table 6). Furthermore, no significant interaction between serum IL-18 levels and less negative GLS were noted in terms of all-cause mortality (*p* = 0.74). Receiver operating characteristic curve analysis revealed that using a combination of GLS and IL-18 may facilitate risk stratification for the outcomes (Figure 3).

**Table 4:** Univariate Cox regression analysis of factors in relation to all-cause mortality.

Variables	All-Cause Mortality	
	HR (95% C.I.)	*p* value
Gender, male	0.60 (0.23–1.56)	0.29
Age (years)	1.05 (1.002–1.10)	0.04
Prevalent HF	1.19 (0.34–4.15)	0.78
Prevalent CAD	2.76 (1.05–7.27)	0.04
Hypertension	3.67 (1.35–9.97)	0.01
Diabetes mellitus	1.89 (0.70–5.12)	0.21
LV hypertrophy	0.31 (0.09–1.10)	0.07
Hyperlipidemia	0.54 (0.16–1.87)	0.33
Chronic hepatitis	1.87 (0.69–5.05)	0.22
Hemodialysis duration (years)	0.97 (0.86–1.09)	0.59
Kt/V	0.47 (0.05–4.24)	0.50
IDWG (%)	1.02 (0.79–1.33)	0.86
Heart rate (beats/minute)	0.99 (0.95–1.04)	0.75
CCB prescription	0.54 (0.21–1.43)	0.22
β-blockade prescription	0.63 (0.23–1.70)	0.36
ACEIs/ARBs prescription	0.29 (0.10–1.18)	0.12
Statin prescription	0.66 (0.15–2.89)	0.58
Serum Albumin (g/dL)	0.07 (0.01–0.36)	0.002
Serum hs-CRP (mg/dL)	1.25 (0.92–1.49)	0.11
Serum IL-6 (pg/ml)	0.99 (0.94–1.05)	0.83
Serum IL-18 (pg/ml)	1.001 (1.00–1.001)	0.007
Serum PICP (ng/ml)	1.00 (0.99–1.002)	0.62
Serum Ca * P	0.98(0.95–1.02)	0.35
LV mass index (gm/m^2^)	1.00 (0.99–1.01)	0.98
LV EF (%)	0.99 (0.94–1.05)	0.84
LAVi (ml/m^2^)	1.03 (0.96–1.10)	0.47
Average E/e′	0.99 (0.93–1.06)	0.75
GLS (%)	1.09 (0.95–1.24)	0.23
LSRs (sec^−1^)	1.66 (0.16–17.42)	0.67
Circumferential strain (%)	1.06 (0.96–1.16)	0.25
Systolic circumferential strain rate (sec^−1^)	1.09 (0.43–2.78)	0.85
Increased IL-18 level (≥654.2 pg/ml)	2.71 (1.01–7.70)	0.04
Less negative GLS (>−15%)	2.61 (0.95–7.18)	0.05
Less negative Circumferential strain (>−23.3%)	1.50 (0.40–5.65)	0.55

Abbreviations: ACEI, angiotensin-converting enzyme inhibitor; ARB, angiotensin II-receptor blocker; CAD, coronary artery disease; CCB, calcium channel blocker; hsCRP, high-sensitivity C-reactive protein; IDWG, inter-dialytic weight gain; IL, interleukin; Kt/V, dialysis clearance; LV, left ventricular; PICP procollagen type I C-terminal peptide; EF, ejection fraction; E/e′, early transmitral velocity to tissue Doppler mitral annular early diastolic velocity ratio; GLS, global left ventricular peak systolic longitudinal strain; LAVi, left atrial volume index; LSRs, systolic longitudinal strain rate; LSRe, early diastolic longitudinal strain rate.

**Table 5:** Correlation study between different parameters.

	Albumin	IL-18	hsCRP	Ca*P	HD duration (years)	
GLS	Pearson/Spearman's★ Correlation	−0.174	0.196★	0.199★	−0.089	−0.191★
	*p* value	0.15	0.10★	0.09★	0.46	0.13★
CS	Pearson/Spearman's★ Correlation	−0.217	0.239★	0.188★	−0.021	−0.140★
	*p* value	0.09	0.07★	0.14★	0.87	0.30★
Albumin	Pearson/Spearman's★ Correlation		−0.196★	−0.549★	0.197	−0.061★
	*p* value		0.10★	<0.01★	0.10	0.63★
IL-18	Pearson/Spearman's★ Correlation	−0.196★		0.281★	0.027★	0.218★
	*p* value	0.10★		0.02★	0.82★	0.08★
hsCRP	Pearson/Spearman's★ Correlation	−0.549★	0.281★		0.013★	0.192★
	*p* value	<0.01★	0.02★		0.91★	0.13★
Ca*P	Pearson/Spearman's★ Correlation	0.197	0.027★	0.013★		0.100★
	*p* value	0.10	0.82★	0.91★		0.43★

Abbreviations: CS, circumferential strain; GLS, Global left ventricular peak systolic longitudinal strain; HD, hemodialysis; hsCRP, high-sensitivity C-reactive protein; IL, interleukin.

^★^ The non-normal distributed data were analyzed by Spearman's correlation analysis.

**Table 6:** Multivariate Cox regression analysis for all-cause mortality in stable hemodialysis patients.

	Model 1		Model 2		Model 3		Model 4		Model 5	
Covariates	HR (95% CI)	*p*	HR (95% CI)	*p*	HR (95% CI)	*p*	HR (95% CI)	*p*	HR (95% CI)	*p*
Age	1.003 (0.95–1.06)	0.91	1.01 (0.96–1.07)	0.6	1.02 (0.97–1.07)	0.43	1.02 (0.97–1.09)	0.44	1.04 (0.98–1.09)	0.2
Serum albumin	0.06 (0.01–0.32)	0.001	0.11 (0.02–0.53)	0.006	0.08 (0.02–0.39)	0.002	0.2 (0.04–0.95)	0.04	0.13 (0.03–0.64)	0.01
Prevalent CAD	2.54 (0.91–7.05)	0.07	–	–	–	–	–	–	–	–
Hypertension	–	–	3.31 (1.11–9.82)	0.03	3.78 (1.29–11.12)	0.02	4.59 (1.46–14.4)	0.01	4.70 (1.56–14.13)	0.01
Less negative GLS(>−15%)	–	–	–	–	–	–	3.55 (1.18–10.7)	0.02	4.15 (1.31–13.21)	0.01
Serum IL-18	–	–	–	–	1.001 (1.00–1.001)	0.02	–	–	1.001 (1.00–1.001)	0.01

Abbreviations: CAD, coronary artery disease; CI, confidence interval; GLS, global left ventricular peak systolic longitudinal strain; HR, hazard ratio; IL, interleukin.

–, not enrolled.

## Discussion

In this prospective cohort study, we demonstrated for the first time that hemodialysis patients with high serum IL-18 concentrations had worse LV systolic function, represented as less negative GLS and CS (Table 2), and higher all-cause mortality (Figure 1A) compared with patients with low IL-18 levels. However, increased IL-18 levels predicted higher all-cause mortality in clinically stable hemodialysis patients, independently of LV dysfunction (Tables 4 and 6). Furthermore, we highlighted the concomitant application of GLS and IL-18 for better prognostic risk assessment in clinically stable hemodialysis patients (Table 6 and Figure 3).

Chiang et al. [7] reported that serum IL-18 level is a strong predictor of hospitalization in hemodialysis patients; however, serum IL-18 did not predict future mortality in hemodialysis patients in their studies. The follow-up period in their study was 12 months, which may be too short when evaluating mortality (only 10.8% in their study). On the contrary, the follow-up period in our study was 31months (26.4±9.4 months in the enrolled patients) with 22.7% mortality, and we showed that increased IL-18 levels predicted all-cause mortality in stable hemodialysis patients.

Malnutrition-inflammation-atherosclerosis (MIA) syndrome has been shown to be a powerful prognostic indicator in hemodialysis patients [32], [33]. Although albumin has been proven to be a strong predictor of mortality in ESRD patients [34], [35], the correlation between albumin and pro-inflammatory cytokines levels is still under debate [7], [33]. Our data showed that that there was no significant difference in albumin levels between the low and high IL-18 groups; however, a significant difference was noted in the hsCRP levels (Table 1). Furthermore, IL-18 levels, as a continuous variable, showed modest correlations with hsCRP levels (Table 4), suggesting that IL-18 may play a role in the MIA syndrome of ESRD patients.

Compared with healthy subjects, ESRD patients commonly have elevated IL-18 levels, which might represent of a subclinical-inflammation state in stable HD patients [7], [8]. Both uremic milieus and dialysis-related factors (such as bio-incompatibility with the dialyzer or chronic endotoxin exposure) may contribute to the high IL-18 levels [7]. There might be a connection between IL-18 levels and chronic hepatitis in stable hemodialysis patients (IL-18 levels with *vs.* without hepatitis: 1172.39±862.28 *vs.* 731.64±550.49 pg/ml, *p* = 0.01). However, chronic hepatitis is not a prognostic predictor (Table 4).

IL-18 is a middle-molecule and protein-bound uremic toxin, which is difficult to remove by any of the currently available dialytic strategies [36]. Uremic toxins affect many organs, including cardiovascular systems. It is speculated that IL-18 may cause LV dysfunction indirectly by aggravating coronary atherosclerosis or directly by acting on cardiomyocytes to induce myocardial remodeling [11]-[14]. For the vascular effects, IL-18 may stimulate Th1 cells to secrete interferon-γ [9], which plays a pivotal role in inflammation-related vascular injury [10] and intimal atherosclerotic plaque formation and its instability [11]. Clinical observations revealed associations between higher serum concentrations of IL-18 and the carotid intima-media thickness (IMT) [37] and also with poor outcome of CAD patients [15]. For the cardiac effects, IL-18 has been demonstrated to induce cardiac systolic and diastolic dysfunction *in vivo* [13], [14]. There were several possible mechanisms of IL-18 involved myocardial injury. IL-18 may enhance activation of Myeloid Differentiation 88 and IL-1R associated kinase pathways, then up-regulate nuclear factor-κB (NF-κB) and p38 mitogen-activated protein kinases, and finally result in myocardial dysfunction [38]–[40]. Furthermore, after myocardial injury, IL-18 may induce apoptosis by inducing cytokines release and up-regulating either extrinsic death receptor signaling or intrinsic mitochondrial control pathway [41]–[43]. Furthermore, IL-18 was demonstrated to stimulate phosphatidylinositol 3-kinase-Akt pathway, followed by activating NF-κB and expressing transcription factor GATA4 and atrial natriuretic peptide [44], [45].

In the present study, most of the hemodialysis patients had LV hypertrophy, high average E/e′ values, and increased PICP levels that were compatible with cardiac fibrosis and diastolic dysfunction [25]. However, both the high and low IL-18 groups had diastolic dysfunction, increased LV filling pressure, and similar severity of cardiac fibrosis, suggesting that IL-18 may not be a major risk factor for LV diastolic dysfunction in hemodialysis patients.

It should be noted that although hemodialysis patients with high IL-18 level tend to have worse LV systolic function compared with patients with low IL-18 levels, there was no significant linear correlation between IL-18 levels and GLS. This finding suggests other factors may play a significant role in systolic dysfunction in hemodialysis patients. There are many potential risk factors other than IL-18 for LV systolic dysfunction in hemodialysis patients, such as hypertension, hyperlipidemia, endothelial dysfunction, oxidative stress, insulin resistance, hemodialysis-related myocardial stunning, intra-dialytic hypotension, hypervolemia and positive sodium balance, high oxidative stress status, abnormalities of mineral metabolism with low 1,25-dihydroxyvitamin D and high parathyroid hormone levels, and high indoxyl sulfate level [41]. Besides, we found that those patients with less negative GLS (>−15%) suffered from background HF and/or CAD more often than patients with more negative GLS (≤−15%). Since multi-variate Cox regression analyses showed that neither CAD nor HF was an independent prognostic predictor in our studied patients, and there is no large difference among hazard ratios of GLS in different models (including CAD and/or HF or not) (Table S1), the prognostic impact of GLS may not be attributable to the background CAD and/or HF. In this study, we clearly demonstrated that both elevated IL-18 levels and less negative GLS predict all-cause mortality independently in stable hemodialysis patients, suggesting the possibility that different mechanisms increase mortality in these two conditions. The reasons for this finding are currently unknown; however, we speculated that high IL-18 levels might result in atherosclerotic plaque progression and increased vulnerability of coronary or other arteries, which might lead to increased mortality. In contrast, less negative GLS is a marker of subclinical LV dysfunction which may progress to overt HF and result in high mortality. This speculation is based on the fact that there has been no human study demonstrating the association between high IL-18 level and HF-related mortality, although animal studies have suggested that high IL-18 may cause LV systolic and diastolic dysfunction. Nevertheless, further studies are needed to delineate this issue.

There were several limitations regarding this study that are worth noting. First, the number of enrolled patients was limited, so this study does not have enough power to explore the association between serum IL-18 levels and all-cause mortality in greater details. For example, we could not explore the role of cardiac troponin T in this association. In addition, we cannot analyze the impact of IL-18 on cause-specific mortality, such as cardiovascular mortality and infection-related mortality. With the current sample size, however, we have enough statistical power to detect the effects of IL-18 on all-cause mortality already, and therefore the finding was statistically significant. Nevertheless, a study with larger sample size to evaluate more predictors simultaneously in the future may help confirm our findings. Second, some hemodialysis patients had severe valvular heart diseases, atrial fibrillation, or poor echocardiographic image quality, which excluded the possibility of GLS analysis. Third, we recognized that although this study was cohort observational study, the laboratory tests and echocardiographic study were performed once in the start of the study. We could not conclude whether longitudinal laboratory tests and echocardiographic study might improve their predictive powers. Fourth, we did not measure brain natriuretic peptide levels. Last, we did not have the IL-18 data for the healthy subjects.

## Conclusions

Hemodialysis patients with high IL-18 level tend to have worse LV systolic function and a higher mortality rate compared with patients with lower IL-18 levels. However, both elevated IL-18 and less negative GLS are independent prognostic predictors in stable hemodialysis patients. The combination of GLS and IL-18 may facilitate risk stratification for the outcomes and inform clinical decision making in stable hemodialysis patients. Further studies for developing strategies to treat hemodialysis patients who have increased IL-18 and/or less negative GLS are warranted.

## Supporting Information

Table S1 **The association of Less negative GLS (>−15%) with all-cause mortality using multivariate Cox regression analysis.**
(DOC)
