# Supplementary material for: Elevated Serum Interleukin-18 Level Is Associated with All-Cause Mortality in Stable Hemodialysis Patients Independently of Cardiac Dysfunction
Source: PLoS One. 2014 Mar 5;9(3):e89457. doi: 10.1371/journal.pone.0089457 (PMC3943737; doi:10.1371/journal.pone.0089457)
Supplement: Table S1 — The association of Less negative GLS (>−15%) with all-cause mortality using multivariate Cox regression analysis. (DOC) [file pone.0089457.s001.doc]

**Supplemental Table S1.** The association of Less negative GLS (>-15%) with all-cause mortality using multivariate Cox regression analysis

| Covariates | Model 1 | | Model 2 | | Model 3 | |
| --- | --- | --- | --- | --- | --- | --- |
|  | HR (95% CI) | *p* | HR (95% CI) | *p* | HR (95% CI) | *p* |
| Age | 1.03 (0.97-1.09) | 0.40 | 1.01 (0.96-1.07) | 0.64 | 1.02 (0.97-1.09) | 0.44 |
| Serum albumin | 0.08 (0.01-0.39) | 0.002 | 0.08 (0.02-0.39) | 0.002 | 0.2 (0.04-0.95) | 0.04 |
| Prevalent CAD | 2.32 (0.67-8.03) | 0.19 | 2.32 (0.67-8.03) | 0.19 | -- | -- |
| Hypertension | 3.85 (1.31-11.3) | 0.01 | 3.85 (1.31-11.3) | 0.01 | 4.59 (1.46-14.4) | 0.01 |
| Heart failure | 3.44 (0.62-19.1) | 0.16 | -- | -- | -- | -- |
| Less negative GLS (>-15%) | 3.63 (1.21-10.9) | 0.02 | 3.63 (1.21-10.9) | 0.02 | 3.55 (1.18-10.7) | 0.02 |

Abbreviations: CAD, coronary artery disease; CI, confidence interval; GLS, global left ventricular peak systolic longitudinal strain; HR, hazard ratio.
